# Methyl Jasmonate-Induced Monoterpenes in Scots Pine and Norway Spruce Tissues Affect Pine Weevil Orientation

**DOI:** 10.1007/s10886-016-0790-z

**Published:** 2016-11-28

**Authors:** Lina Lundborg, Göran Nordlander, Niklas Björklund, Henrik Nordenhem, Anna-Karin Borg-Karlson

**Affiliations:** 1 Department of Chemistry, Organic Chemistry, KTH Royal Institute of Technology, School of Chemical Science and Engineering, SE-100 44 Stockholm, Sweden; 2 Department of Ecology, Swedish University of Agricultural Sciences, P.O. Box 7044, SE-750 07 Uppsala, Sweden; 3 Institute of Technology, Division of Organic Chemistry, Tartu University, 50411 Tartu, Estonia

**Keywords:** *Hylobius abietis*, *Pinus sylvestris*, *Picea abies*, Monoterpenes, Methyl jasmonate, Enantiomers

## Abstract

In large parts of Europe, insecticide-free measures for protecting conifer plants are desired to suppress damage by the pine weevil *Hylobius abietis* (L.). Treatment with methyl jasmonate (MeJA), a chemical elicitor already used in crop production, may enhance expression of chemical defenses in seedlings in conifer regenerations. However, in a previous experiment, MeJA treatment resulted in substantially better field protection for Scots pine (*Pinus sylvestris* L.) than for Norway spruce (*Picea abies* (L.) Karst.). Hypothesizing that the variations may be at least due partly to volatiles released by MeJA-treated seedlings and their effects on pine weevil orientation, we examined tissue extracts of seedlings (from the same batches as previously used) by two-dimensional GC-MS. We found that the MeJA treatment increased contents of the monoterpene (*−*)-β-pinene in phloem (the weevil’s main target tissue) of both tree species, however, the (*−*)-β-pinene/(*−*)-α-pinene ratio increased more in the phloem of *P. sylvestris*. We also tested the attractiveness of individual monoterpenes found in conifer tissues (needles and phloem) for pine weevils using an arena with traps baited with single-substance dispensers and pine twigs. Trap catches were reduced when the pine material was combined with a dispenser releasing (*−*)-β-pinene, (*+*)-3-carene, (*−*)-bornyl acetate or 1,8-cineole. However, (*−*)-α-pinene did not have this effect. Thus, the greater field protection of MeJA-treated *P. sylvestris* seedlings may be due to the selective induction of increases in contents of the deterrent (*−*)-β-pinene, in contrast to strong increases in both non-deterrent (*−*)-α-pinene and the deterrent (*−*)-β-pinene in *P. abies* seedlings.

**Electronic supplementary material:**

The online version of this article (doi:10.1007/s10886-016-0790-z) contains supplementary material, which is available to authorized users.

## Introduction

The pine weevil [*Hylobius abietis* (L.)] is a major forest pest in Europe and Asia as it feeds on the bark of newly planted conifer seedlings, and may kill up to 90 % of seedlings at some sites without protective measures (Petersson and Orlander 2003). Strong demands for non-insecticide control measures that are not harmful to either ecosystems or workers (FSC 2014; Långström and Day 2004) have led to the development of diverse methods to protect seedlings against pine weevils, including (for instance) use of various feeding barriers (Nordlander et al. 2009), antifeedants (Sunnerheim et al. 2007), and entomopathogenic nematodes and fungi (Williams et al. 2013). The alternative to use agents that trigger plant defenses against pests are applied at commercial scales to protect certain agricultural and horticultural crops (Reglinski et al. 2014). However, this strategy is not currently used to protect forestry plants, but has shown promising potential in recent research (Reglinski et al. 2015; Zas et al. 2014).

Methyl jasmonate (MeJA) has been used in many studies to activate production of defense chemicals in conifers and reduce insect feeding (Fedderwitz et al. 2016; Krokene 2015). Methyl jasmonate elicitation influences the plants’ metabolism (Reymond and Farmer 1998), inter alia triggering the production of terpene and phenolic defenses (Fäldt et al. 2003; Huber et al. 2005; Keeling and Bohlmann 2006; Martin et al. 2002). It also induces formation of traumatic resin ducts and polyphenolic parenchyma cells (Hudgins et al. 2004). Partly because of their potential applications in pest control, physiological and chemical responses to MeJA have been widely studied in both conifer seedlings (Fäldt et al. 2003; Gould et al. 2008; Heijari et al. 2005; Moreira et al. 2009, 2012; Pham et al. 2014; Zeneli et al. 2006) and trees (Erbilgin et al. 2006; Fäldt et al. 2003; Schiebe et al. 2012; Zhao et al. 2011).

Chemical defenses of conifers vary according to species (Keeling and Bohlmann 2006), age and tissue (Kännaste et al. 2013a). Genotypic variants within species of conifers, known as chemotypes, also may have different profiles of defense-related volatiles and other compounds (Hanover 1992; Kännaste et al. 2013b). (*+*)-3-Carene and (*+*)-α-pinene are the main volatiles in tissues of Scots pine (*Pinus sylvestris* L.) (Sjödin et al. 2000), while those of Norway spruce (*Picea abies* (L.) Karst) are (*−*)-α-pinene, (*−*)-β-pinene, and a few other monoterpenes, e.g., (*−*)-limonene and (*−*)-β-phellandrene (Silvestrini et al. 2004; Sjödin et al. 2000).

Pine weevil populations frequently are high in areas that are regenerated by clear-cutting since that provides a high and constant supply of fresh stumps and roots that are suitable breeding substrates (Björkman et al. 2015). Pine weevil is a polyphagous insect that feeds mainly on tender bark of roots and branches of mature trees and also on various field-layer vegetation (Orlander et al. 2001; Wallertz et al. 2006). Thus, despite that it is feeding on planted conifer seedlings, which makes it an economically important pest, seedlings only constitute a minor part of their diet on planted clear-cuts (Bylund et al. 2004). Pine weevils use the volatiles emitted by conifer seedlings to find them (Björklund et al. 2005). Previously attacked seedlings with feeding scars are more attractive to pine weevils than unattacked seedlings (Nordlander 1991). *Picea abies* seedlings that are a few weeks old mainly emit green leaf volatiles (Pettersson et al. 2008), which do not attract weevils. Thus, these seedlings may pass undetected. When older, their bouquet shifts mainly to mono- and sesqui-terpenes (Pettersson et al. 2008), and seedlings usually are taken from nurseries and established in the field at an age of 1–3 yr. (Hallsby 2013). The pine weevil also has been observed to feed on needles (Fedderwitz et al. 2014), which may activate inducible defenses. Terpene defense chemicals that are produced in a tissue upon attack may be reallocated to other tissues as part of an induced defense system (Heijari et al. 2005).

A previous field study showed that MeJA treatment increased non-volatile quantitative defenses in stems (non-volatile resin) and needles (non-volatile resin and total phenolics) of both *P. sylvestris* and *P. abies* seedlings (Zas et al. 2014). However, it had much greater protective effects, in terms of reductions of debarked area and seedling mortality, in the *P. sylvestris* seedlings (Zas et al. 2014). In the complementary study reported here, we analyzed volatiles in seedlings from the same batches of MeJA-treated and control seedlings to evaluate the constitutive and MeJA-induced volatile profiles of tissues of both species’ seedlings. We also tested the potential pine weevil-deterring effects of the main volatiles by adding them to pine material in traps. The resulting effects on catches, and other reported findings, provide indications of major determinants of the effectiveness of MeJA treatment.

## Methods and Materials

### Seedling Material

Seedlings were from the same batches as those used in the companion study Zas et al. (2014). Briefly, one-yr.-old *P. sylvestris* and *P. abies* seedlings grown from seeds of a central Swedish origin in 50 cm^3^ containers were obtained from a commercial nursery (Sjögränd nursery, Bergvik Skog AB, Uddeholm, Sweden). In spring 2011, sets of the seedlings were sprayed with 2.5 % EtOH in deionized water containing emulsified MeJA (Sigma Aldrich ref. 39,924–52-2) at 5, 10, or 25 mM. Controls received the EtOH solution with no MeJA, and the treatments were applied twice, two and four wk. after the seedlings had been taken out from over-winter cold storage. After another 5 wk., on July 12 to 13, 2011, seedlings subjected to all treatments were cut by the root, and the needle and stem tissues were preserved in a − 80 °C freezer.

### Chemical Analysis of Volatiles

**Fig. 1:**
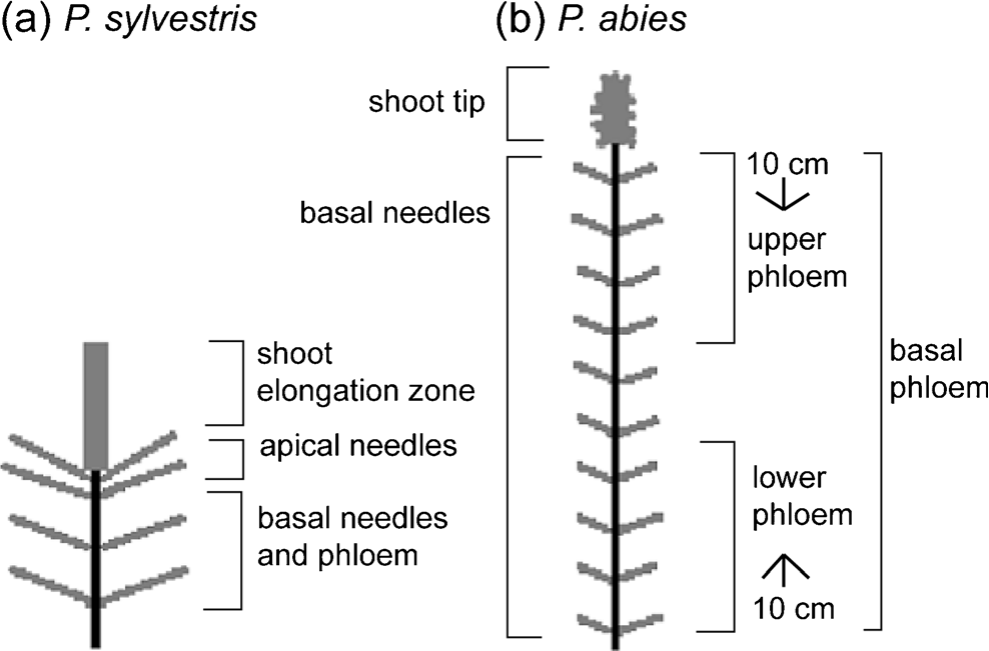
Scheme of chemical sampling of tissues of **a** *Pinus sylvestris* and **b** *Picea abies* seedlings

Seedlings treated with no MeJA were used to analyze constitutive volatiles of seedlings of the two conifer species, and those treated with the highest concentration (25 mM) were selected to analyze induced volatiles, as Zas et al. (2014) observed a dose-dependent increase of field protection against pine weevil feeding. Tissues from sets of eight *P. sylvestris* seedlings were prepared for this purpose from September 10 to 15, 2011, and tissues from sets of eight *P. abies* seedlings from October 22 to November 1, 2012. The *P. sylvestris* samples included basal needles, basal phloem, apical needles, and shoot elongation zone, defined here as the part of the shoot above the apical node (Fig. 1). Phloem samples were taken from a section of the stem extending at most ca. 5 cm upwards from just above the first node. Avoiding notches, all available phloem tissue in these sections was used. The shoot elongation zone of the *P. sylvestris* seedlings had not yet differentiated into phloem and xylem. For *P. abies*, samples were taken from three ‘basal’ tissues (10 cm of lower phloem, 10 cm of upper phloem and needles) and the shoot tip, as illustrated in Fig. 1. The stems were approx. 25 cm tall, so almost all phloem tissue was used. The shoot tips of the *P. abies* seedlings consisted mainly of needles. Needle samples from these seedlings consisted of a mixture of needles from their entire stems.

The sampled tissues were chopped in 1 mm pieces, and extracted 24 h at room temperature in 4 ml glass vials following the standard protocol (Persson et al. 1993) with 1 ml of hexane containing 0.05 mg pentadecane (Lancaster synthesis, Alfa Aesar) as the internal standard. Extracts were transferred to 2 ml vials and kept in a − 30 °C freezer, until analysis by using a system (Agilent Technologies, CA, USA) consisting of two 7890A gas chromatographs (GCs) coupled to a 5975C mass spectrometer (MS). The first and second GCs were equipped, respectively, with a DB-5 fused silica capillary column and a Cyclodextrin-β column for separation of enantiomers of the chiral monoterpenes (dimensions of both: 30 m, i.d. 0.25 mm, film thickness 0.25 μm; Agilent).

For each analysis, the temperature of the first oven was held at 40 °C for 1 min, increased by 10 °C min^−1^ to 270 °C, held there for 1 min, then increased by 100 °C min^−1^ to 300 °C, and held again for 1 min. Samples (1 μl) were injected splitless at an injector temperature of 240 °C, with a purge time of 1.5 min, and the carrier gas was helium. The mass scanning range of the MS was set at *m/z* 35 to *m/z* 350. Co-elutants β-phellandrene and limonene, both of which yield a main fragment of *m/z* 93, were separated using the *m/z* 68 to *m/z* 93 ratio of limonene analytical standard, as *m/z* 68 is a main fragment of limonene but not present in the β-phellandrene mass spectrum. When 1,8-cineole (present in phloem samples, M = 154) co-eluted with these substances, it was quantified using the *m/z* 154 to *m/z* 93 ratio of 1,8-cineole standard. The identity of (−)-β-phellandrene was confirmed by analysis using the second column with an extract of lodgepole pine (*Pinus contorta* Dougl.) as a natural reference, in which (−)-β-phellandrene is a major compound. Unknown compounds were tentatively identified using NIST Mass Spectral Library matches in G1701EA MSD ChemStation software (also from Agilent), and available standards.

The temperature of the second oven was kept at 55 °C for 0.1 min then increased by 1 °C min^−1^ to 78 °C, held for 0.1 min, then increased by 100 °C min^−1^ to 200 °C, which was held for 1 min. The transfer line was set to 50 °C for 10 min, increased by 10 °C min^−1^ to 130 °C and held for 8 min. α-Pinene, β-pinene, and limonene were transferred at 5.6–6.1, 6.3–6.8, and 7.4–7.9 min, respectively, to the second column for separation of their stereoisomers.

There are differences both among tissues and between species in proportions of the enantiomers of the main monoterpene α-pinene in volatile contents of *P. sylvestris* and *P. abies* seedlings. In addition, the pine weevil is known to respond differently to enantiomers of α-pinene and limonene (Wibe et al. 1998), so both α-pinene and limonene were selected for chiral analysis. β-Pinene is dominated by (*−*)-β-pinene in conifer tissue extracts (Persson 2003), but its elution time was sufficiently distinct to allow easy separation from the other monoterpenes in the same run. The major constituent 3-carene was not analyzed chirally, but only the (*+*)-enantiomer is produced (Silvestrini et al. 2004; Sjödin et al. 1996, 2000) so this was unnecessary.

### Bioassay of Pine Weevil Responses

A circular (1 m diam), multi-choice arena with 16 traps was used to examine pine weevil responses to tested substances, alone and in combination with odor from pieces of *P. sylvestris* twigs, as described in detail by Azeem et al. (2013). The top of the arena was open, and the ventilation system sucked air out of the room above it. Traps containing the odor treatment were capped but had eight 14-mm diam holes situated around the circumference, with their lower edge 5 mm above the arena floor. Thus, the weevils had to actively enter the traps through these holes. The 16 traps in the arena provided four randomly assigned replicates of four treatments, consisting of additions of: 1) *P. sylvestris* twig, 2) test substance + *P. sylvestris* twig, 3) the test substance, and 4) nothing (empty trap). Substances were released from 1.5 ml micro tubes with a 9 mm opening (Sarstedt, ref. 72.688). The pieces of twigs used in assays for each substance were collected from a single tree and were of similar size (length ca. 20 mm, diam ca. 8 mm). One twig from each tree was used to assess the chemotypes of the trees from which the twigs were gathered (Supplementary Fig. 1). Each run of the bioassay lasted 18 h and included the introduction of one terpene to 50 weevils of one sex, and was replicated five times for each sex separately. Between runs, the arena and traps were cleaned and treatments (dispensers with test substances and pine twigs) were changed and randomly assigned to the traps.

Chemicals introduced to the weevil were analytical standards of (*−*)-β-pinene, (*−*)-α-pinene, (*+*)-3-carene, and (*−*)-bornyl acetate, with purities according to the commercial source (Sigma Aldrich), for sums of enantiomers, ≥ 98.5, ≥ 99.0, ≥ 98.5, and ≥99.0 %, respectively. Our GC-GC-MS separation of (*−*)-β-pinene and (*−*)-α-pinene showed enantiomeric purities of 98 and 99 %, respectively. 1,8-Cineole and terpinolene were technical standards for GC analysis (purity ~98 % and ≥85 %). Only the (*−*)-enantiomer of bornyl acetate is reportedly present in *P. sylvestris* needles (Kännaste et al. 2013a), so only this enantiomer was used. Before every arena bioassay, i.e., bioassay for each replicate and sex, emissions from one dispenser over 22 ± 2 h were quantified from mass losses recorded using a Mettler balance with an accuracy of ±0.1 mg, and the results were used to calculate average emission rates for each test substance in mg h^−1^.

### Statistical Analysis

To visualize correlations between contents of the main monoterpenes in the seedlings with species, tissue and MeJA treatment, dimensions of the dataset acquired from the GC-MS analysis were reduced by Principal component analysis (PCA), using FactoMinerR version 1.26 (Husson et al. 2014) in R version 3.1.0 (the R Foundation for Statistical Computing, Austria), using relative amounts weighted by the sum of monoterpenes included in the model. Arcsine transformation was applied prior to analysis to improve the normality of data distributions. Each compound in the model was tested separately against the first and second principal components to identify influential compounds. Differences in average values for samples grouped by a descriptive variable (seedling identity, tissue or treatment) also were tested with one-way ANOVA. These additional tests (implemented in the FactoMineR package) had no impact on the model, but were included to assist interpretation of the data.

Arithmetic means and standard errors (SE) were calculated to show absolute tissue contents of the measured volatiles. Numbers above tissue contents in the figures are contrasts from Tukey range tests implemented (with a 95 % confidence level) in the “lsmeans” package (Lenth and Hervé 2015) following one-way ANOVA of effects of all treatment and tissue combinations shown. Student’s *t*-test (equal variance, two-tailed) on logarithmic data (to normalize distributions) was used to test the significance of changes in concentration of a volatile in a specific tissue due to MeJA treatment.

Logistic regression was used to evaluate responses of weevils of each sex to each substance in the multi-choice arena tests. Only data from responding weevils were considered, i.e., those that had not made a choice when a bioassay was ended were not included. A generalized linear model was constructed for each volatile-sex combination using the glm function (family = binomial, link function = logit) in R. The response variable was the proportion of the total number of weevils entering a trap (catches/n) as a function of trap treatment (*df* = 16). The data were not overdispersed (0.56–1.78 for all models). For each model, i.e., each volatile, a pairwise comparison (*df* = 1) was made between pine alone and the pine with volatile combination. Contrasts were investigated with a Tukey range test on a 95 % confidence level of the linear model.

## Results

### Species and Tissue Differences

**Fig. 2:**
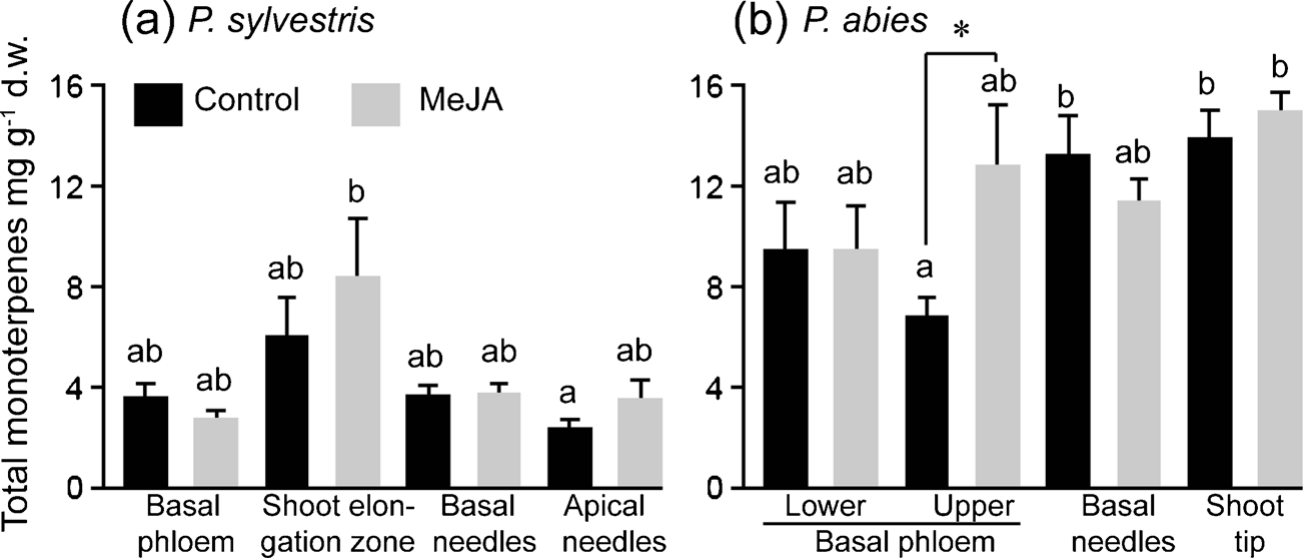
Monoterpene contents of basal and apical tissues of **a** *Pinus sylvestris* and **b** *Picea abies* seedlings, expressed in absolute amounts of pentadecane equivalents (mg g^−1^ d.w.) + SE. Different letters and the asterisk indicate significant differences according to the Tukey range test (at a 95 % confidence level, *df* = 56) and *t*-tests at *P* < 0.05 (*N* = 16), respectively

**Fig. 3:**
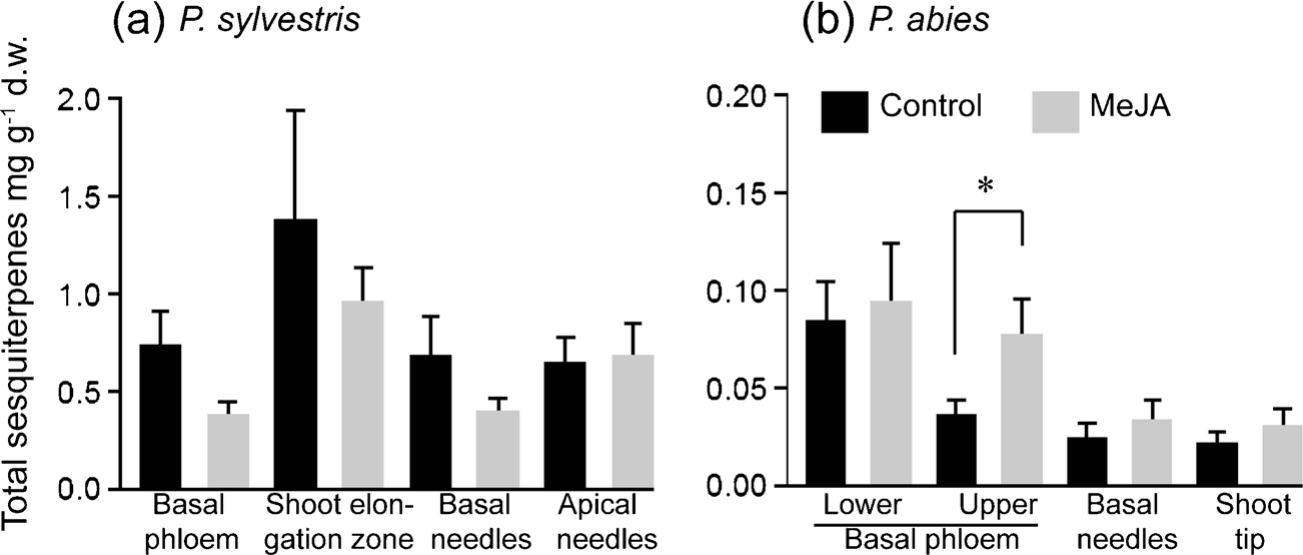
Contents of total sesquiterpenes in tissues of **a** *Pinus sylvestris* and **b** *Picea abies* seedlings, expressed in absolute amounts of pentadecane equivalents (mg g^−1^ d.w.) + SE. The asterisk indicates a significant difference according to a *t*-test at *P* < 0.05 (*N* = 16)

**Fig. 4:**
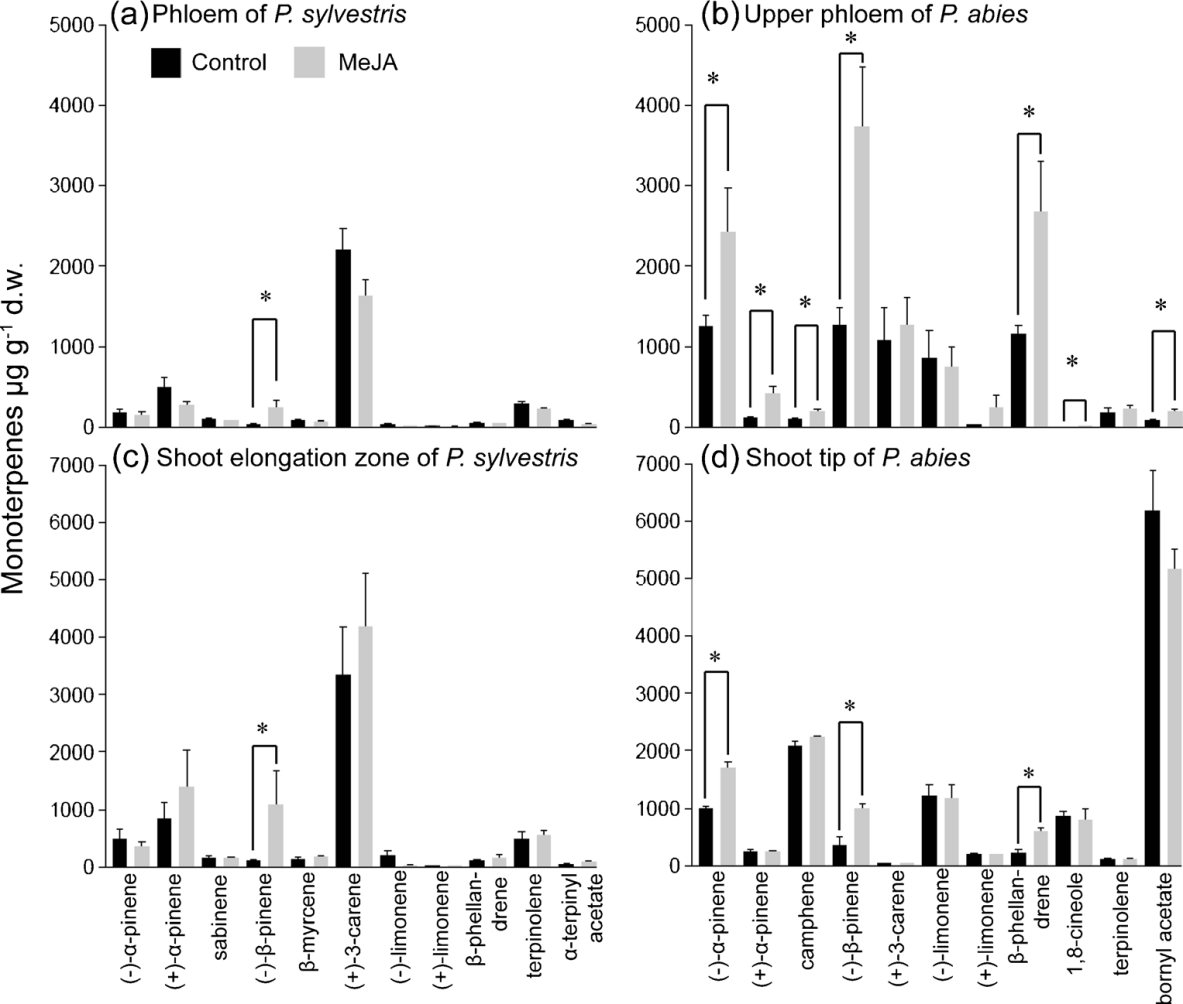
Contents of total monoterpenes in tissues of (**a**, **c**) *Pinus sylvestris* and (**b**, **d**) *Picea abies* seedlings, both basal (a, b) and apical (c, d), expressed in absolute amounts of pentadecane equivalents (μg g^−1^ d.w.) + SE. The asterisks between bars indicate significant differences according to *t*-tests at *P* < 0.05 (*N* = 16)

In the volatile fraction of *P. sylvestris* and *P. abies* tissues, 57 and 46 compounds, respectively, were quantified. These were green leaf volatiles, aromatics, and mono- and sesqui-terpenes (Supplementary Tables 1, 2, 3 and 4). Monoterpenes dominated the volatile fraction of the tissues of both species, and generally were more abundant in *P. abies* than in *P. sylvestris*. The constitutive monoterpene content of the basal phloem of *P. sylvestris* seedlings was 2.6- and 1.9-fold lower than that of the lower and upper basal phloem of *P. abies* seedlings, respectively (Fig. 2). Conversely, sesquiterpenes were, respectively, 11- and 44-fold more abundant in basal phloem of *P. sylvestris* seedlings (Fig. 3). In volatiles of *P. abies*, there were major qualitative differences between the upper phloem and shoot tip (Fig. 4b, d). Monoterpene hydrocarbons dominated the upper phloem and lower phloem, while in the shoot tip and needles the oxygenated monoterpenes bornyl acetate and 1,8-cineole were most abundant (Supplementary Tables 3 and 4).

### Effects of MeJA Treatment on Absolute Amounts of Volatile Contents

In the control seedlings of *P. abies*, the upper phloem contained less monoterpenes than the needles and shoot tip, but in the MeJA-treated seedlings total amounts in these tissues were more similar (Fig. 2b). In the shoot elongation zone of *P. sylvestris*, there were large variations among seedlings, so the mean monoterpene and sesquiterpene contents should be interpreted with caution (Figs. 2a and 3a). In contrast, in *P. abies* seedlings total monoterpene contents in upper phloem increased after the MeJA treatment (Fig. 2b*; N* = 16, *P* = 0.009).

**Fig. 5:**
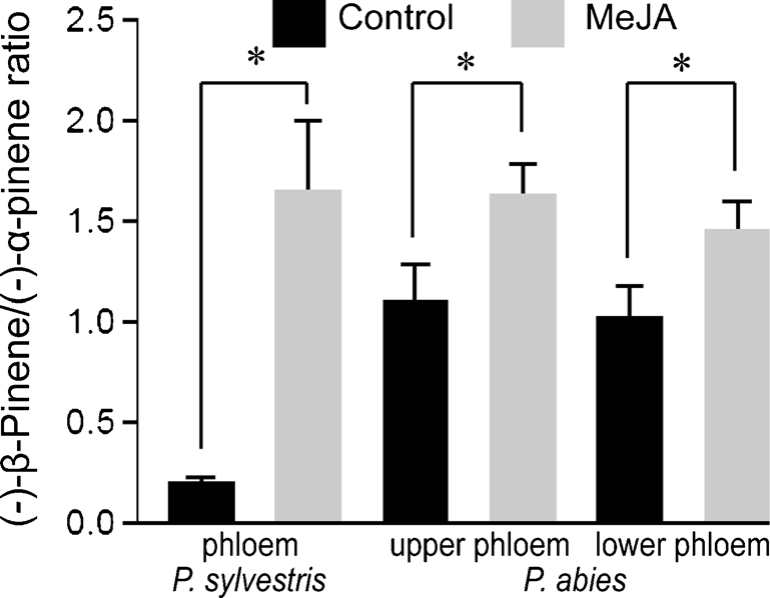
Ratios of (*−*)-β-pinene/(*−*)-α-pinene + SE in basal phloem of control (black) and methyl jasmonate (MeJA)-treated (grey) *Pinus sylvestris* and *Picea abies* seedlings

Responses of both *P. sylvestris* and *P. abies* to the MeJA treatment included increases in amounts of (*−*)-β-pinene in phloem (Fig. 4a, b; *P* = 0.002 and *P* = 0.003, respectively). The higher amounts of (*−*)-β-pinene caused the (*−*)-β-pinene/(*−*)-α-pinene ratio of *P. sylvestris* to become more similar to that of *P. abies* (Fig. 5). In *P. sylvestris* phloem (Fig. 4a), a higher amount of (*−*)-β-pinene was accompanied by a tendency for contents of the main monoterpene (*+*)-3-carene to be lower (*P* = 0.13). MeJA treatment also increased (*−*)-β-pinene contents in the shoot elongation and shoot tip zones (Fig. 4c, d; *P* = 0.02 and *P* = 0.001, respectively). In the shoot tip of *P. abies*, contents of (*−*)-α-pinene (*P* < 0.001) and β-phellandrene (*P* < 0.001) increased (Fig. 4d). In upper phloem of *P. abies*, contents of a number of monoterpenes increased (Fig. 4b), e.g., (*−*)-α-pinene (*P* = 0.04), (*+*)-α-pinene (*P* = 0.02), camphene (*P* = 0.002), and β-phellandrene (*P* = 0.004). In phloem of *P. abies*, the MeJA treatment increased contents of the oxygenated monoterpenes bornyl acetate and 1,8-cineole (Fig. 4b; *P* < 0.001 and *P* = 0.003, respectively). However, we observed no changes in (*−*)-limonene contents, a known pine weevil repellent (Nordlander 1990), in either needles or phloem (Fig. 4).

### Effects of MeJA Treatment on Relative Amounts of Volatile Contents

**Fig. 6:**
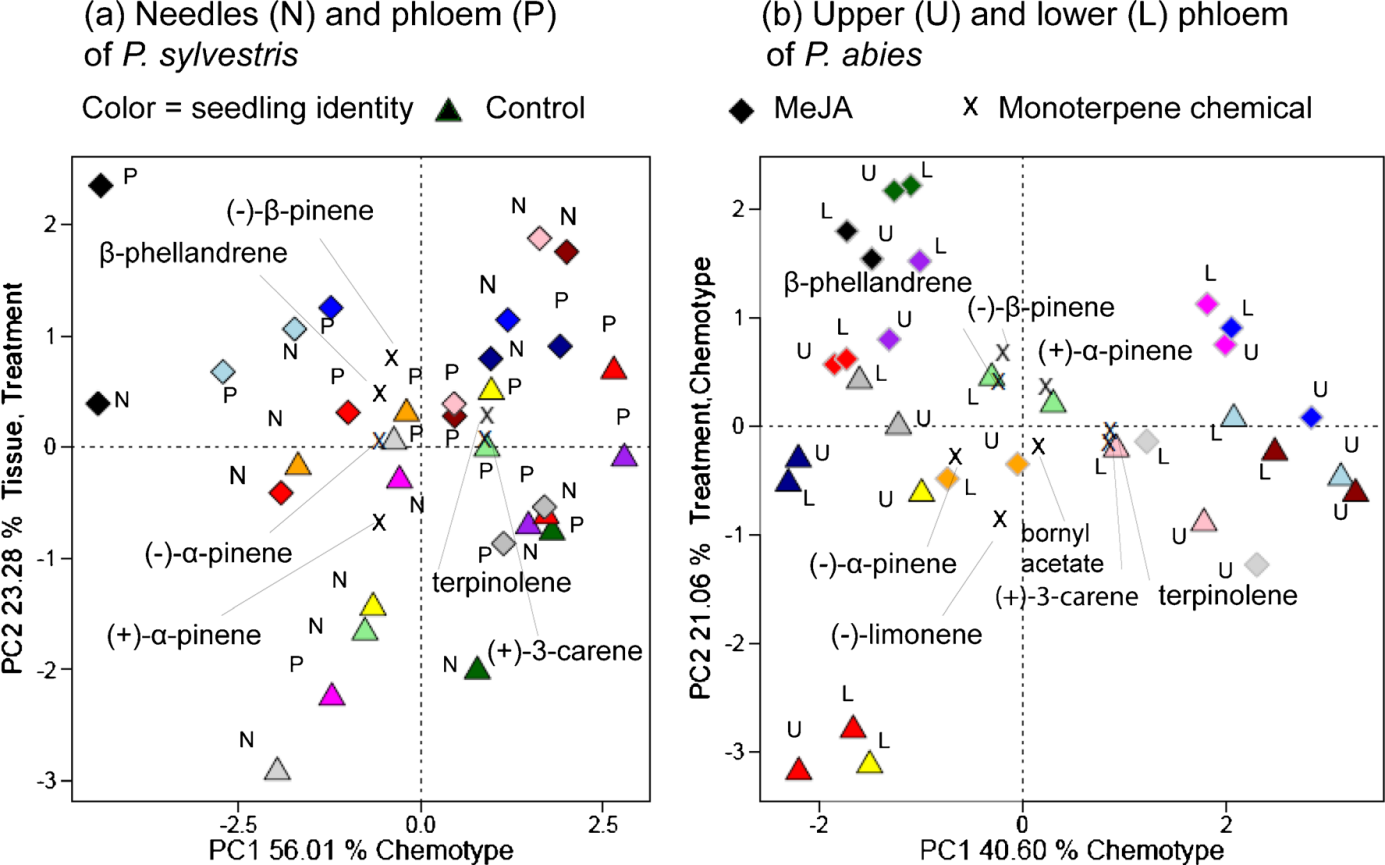
Principal component analysis (PCA) score plots of relative amounts of monoterpenes in sets of eight control and methyl jasmonate (MeJA)-treated **a** *Pinus sylvestris* and **b** *Picea abies* seedlings. Data for individual seedlings are color-coded (color version of the figure is available online), and include contents in needles and phloem tissues of *P. sylvestris*, and phloem tissues (upper and lower) of *P. abies*. Triangles (▲) and diamonds (◆) indicate tissues from control and MeJA-treated seedlings, respectively. Error bars denote SE, and percentages denote percentages of variance explained

Principal component analysis was applied to identify chemotype- and treatment-associated differences in contents of main monoterpenes. In score plots obtained from analyses of *P. sylvestris* phloem and needles, chemotype (seedling identity) had the greatest impact on samples’ positions along the first axis (Fig. 6a), while MeJA treatment only influenced their positions on the second axis, due to increased proportions of (*−*)-β-pinene and β-phellandrene (Fig. 6a). Tissue also influenced positions on the second axis (Fig. 6a). Similarly, PCA showed that chemotype explained most of the variation in analyte contents of *P. abies* phloem samples, notably their (*−*)-α-pinene and (*+*)-3-carene contents (Fig. 6b). Again, MeJA treatment influenced only their positions along the second axis, for instance treated seedlings had higher proportions of (*−*)-β-pinene (Fig. 6b). Positions of the two phloem samples from each *P. abies* seedling also were very close to each other, showing that chemotype and treatment effects on proportions of the main monoterpenes in lower and upper phloem were similar, although a quantitative increase in total monoterpenes was detected only in the upper phloem.

### Bioassay in Multi-Choice Arena

**Fig. 7:**
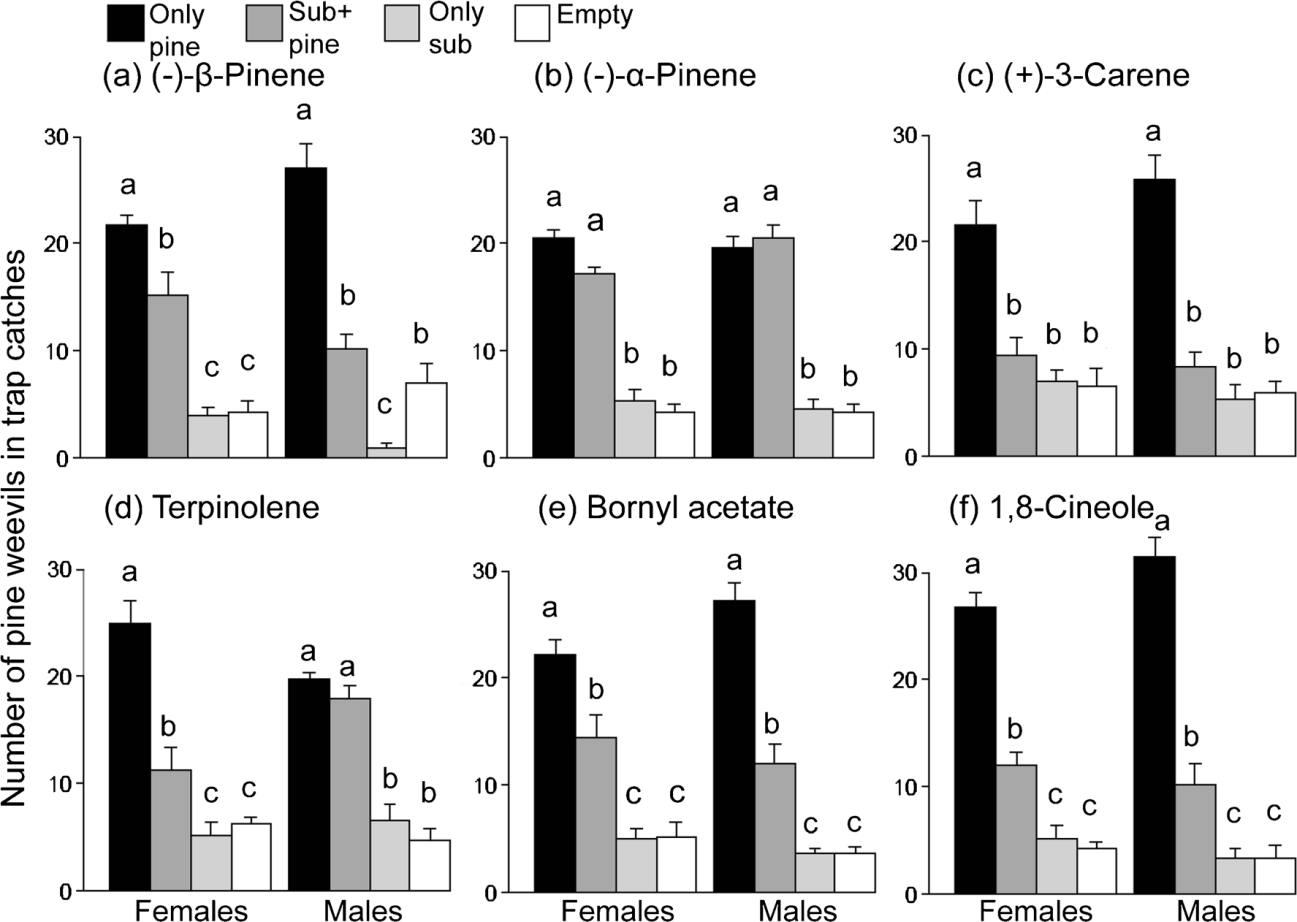
Catches of pine weevils in traps baited with pine twigs (pine) selected volatiles (sub) alone and together, and empty controls, in a multi-choice arena bioassay. Error bars denote SE. Different letters indicate significant differences according to the Tukey range test at a 95 % confidence level

The attraction of pine weevils of both sexes to pine twig odor was significantly reduced by four of the tested compounds: (*−*)-β-pinene (0.47 mg h^−1^), (*+*)-3-carene (1.1 mg h^−1^), (*−*)-bornyl acetate (0.03 mg h^−1^), and 1,8-cineole (0.42 mg h^−1^) (Fig. 7a, c, e and f). Females’ attraction to pine odor also was reduced by terpinolene (0.15 mg h^−1^) (Fig. 7d). In contrast, (*−*)-α-pinene (0.45 mg h^−1^) did not influence trap catches, either in combination with pine odor or alone (Fig. 7b). Catches in traps with all substances except (*+*)-3-carene were higher when they were presented together with pine odor than when presented alone (Fig. 7). The odor from all of the pine twigs used in the analyses was dominated by (*+*)-3-carene, with varying amounts of other monoterpenes (Supplementary Fig. 1). Thus, none of the analyzed pine twigs were of a clearly deviating chemotype. Numbers of non-responding weevils left in the arena at the end of the bioassays varied from two to six.

## Discussion

Changes in volatile terpene contents of tissues of *P. sylvestris* and *P. abies* seedlings induced by MeJA treatment may affect their attractiveness to pine weevils. In the companion study, the applied MeJA treatment reduced pine weevil damage substantially more effectively among *P. sylvestris* seedlings than among *P. abies* seedlings (Zas et al. 2014). Results presented here show that the treatment induced qualitatively different changes in the volatile contents of phloem of *P. sylvestris* and *P. abies* seedlings of the same batches. It induced increases in contents of several monoterpenes in *P. abies* seedlings, but no clear changes in relative proportions of volatiles. In contrast, MeJA induced substantial increases in relative proportions rather than absolute amounts of monoterpenes in the *P. sylvestris* seedlings.

For *P. abies*, the MeJA treatment increased the total amounts of mono- and sesqui-terpenes in the upper phloem. Accordingly, Moreira et al. (2009) and Sampedro et al. (2011) found increased levels of non-volatile resins (diterpene acids) in upper stem tissues of MeJA-treated maritime pine (*Pinus pinaster* Ait.). Moreover, Moreira et al. (2009) observed this induction in upper stem of *P. pinaster* after MeJA treatment of the lower stem. In view of our results for *P. abies*, if pine weevil feeding on lower stem parts similarly enhances upper stem defenses, this might not provide protection against girdling of the lower stem, where girdling most frequently occur in the field.

For *P. sylvestris*, the MeJA treatment did not induce an increase of the total monoterpene contents of the basal phloem. This result is in agreement with a previous study by Heijari et al. (2005). Thus, the greater effectiveness in the field of induced *P. sylvestris* defenses recorded by Zas et al. (2014) indicates that *P. sylvestris* deploys defenses more efficiently. For instance, weaker odor of *P. sylvestris* than of *P. abies* seedlings could contribute to reductions in attractiveness to pine weevils in the presence of alternative food sources.

Both *P. sylvestris* and *P. abies* responses to MeJA included accumulation of (*−*)-β-pinene in phloem, a stress response observed in several conifer species to various treatments (Gould et al. 2009; Moreira et al. 2013; Persson 2003; Sampedro et al. 2010). In *P. abies*, (*−*)-α-pinene was induced at the same time as (*−*)-β-pinene, possibly due to activation of (*−*)α/β-pinene synthase (Martin et al. 2004). In previous work, Joó et al. (2011) detected increases in β-pinene/α-pinene ratios in emissions from Douglas fir (*Pseudotsuga menziesii* (Mirb.) Franco) in response to biotic stress. Accordingly, Sampedro et al. (2010) observed a shift from α-pinene to β-pinene production after MeJA treatment of *P. pinaster* plants. In our bioassay of pine weevil responses, the main monoterpenes (*−*)-α-pinene and (*−*)-β-pinene were presented to the weevil at the same emission rate, and only (*−*)-β-pinene was found to be deterrent. It would be interesting to see if the high (*−*)-β-pinene/(*−*)-α-pinene ratio in induced seedlings was the main mechanism that protects pines from pine weevil attack. However, in stems of *P. abies* and other conifer species, (*−*)-α-pinene and (*−*)-β-pinene are constitutively dominating compounds, which may make treatment effects difficult to distinguish from chemotypical variation.

Increases in non-volatile resin contents in the stems of *P. sylvestris* and *P. abies* seedlings of the same origins, treated in the same way and at the same time as ours were detected by Zas et al. (2014). We found here that the treatment induced quantitative increases in total mono- and sesqui-terpene contents in *P. abies* upper phloem but not in *P. sylvestris* phloem. The induction of diterpene acids, but not monoterpenes, could be due to upregulation of the methyl erythritol 4-phosphate pathway, according to a proteomic study of *P. abies* (Zulak et al. 2009). The monoterpenes also share the geranyl diphosphate precursor (Zulak et al. 2009), which may redirect metabolic fluxes from (*+*)-3-carene to (*−*)-β-pinene production, although we only observed weak, non-significant reductions in (*+*)-3-carene contents in response to MeJA treatment.

In our study, (*+*)-3-carene was the only compound that masked the pine twigs from the pine weevil, so its specific effects could not be distinguished from its effects in combination with the twigs. (+)-3-Carene is reported as a resistance marker for both *P. sylvestris* against pine sawfly [*Diprion pini* (L.)] (Pasquier-Barre et al. 2001) and for Sitka spruce (*Picea sitchensis* (Bong.) Carr.) against white pine weevil [*Pissodes strobi* (Peck.)] (Robert et al. 2010). On the other hand, both (−)-β-pinene and (+)-3-carene seems to have opposite effects to red turpentine beetle (*Dendroctonus valens* Erichson), in the system with the Chinese host tree *Pinus tabuliformis* where they act as attractants (Yan et al. 2005). In our study, (*+*)-3-carene was a main monoterpene of *P. sylvestris* and a minor monoterpene of *P. abies* seedlings. Furthermore, both non-induced and MeJA-treated seedlings with high proportions of (*+*)-3-carene tended to group close to each other in the PCA score plots. Thus, plants with constitutively high (*+*)-3-carene levels may have weaker inducible defenses than other conspecifics because they are already relatively well defended. However, further studies are needed to verify this.

Pine weevils also have been observed previously feeding on *P. abies* needle tissues (Fedderwitz et al. 2014). We found that the oxygenated monoterpenes bornyl acetate and 1,8-cineole were the main volatiles in *P. abies* needles, and that MeJA induced minor increases in their contents in *P. abies* phloem. Interestingly, Andersson et al. (2010) found that 1,8-cineole masked the effects of the aggregation pheromone component, (*4S*)-*cis*-verbenol, of the spruce bark beetle *Ips typographus* (L.), which could for high amounts of 1,8-cineole in *P. abies* trees affect *I. typographus* host selection. Releases of volatiles from feeding scars on stems of *P. sylvestris* reportedly attract weevils (Nordlander 1991), but our results show that emissions of bornyl acetate and 1,8-cineole although deterrent, do not completely mask the presence of pine twig emissions.

Planted seedlings comprise a minor fraction of pine weevils’ diets, and almost all methods for managing this pest, including MeJA treatments, rely on redirecting their feeding from planted seedlings to other food resources on the clear-cuts (Bylund et al. 2004; Nordlander et al. 2009). In the previous experiment, MeJA treatment resulted in substantially better field protection for *P. sylvestris* than for *P. abies* (Zas et al. 2014). The results of the current study indicate that the enhanced field protection of MeJA-treated *P. sylvestris* seedlings may be due to the selective induction of the deterrent (*−*)-β-pinene in the phloem, in contrast to the increases in both (*−*)-β-pinene and the non-deterrent (*−*)-α-pinene in *P. abies* seedlings.

## Electronic supplementary material

ESM 1 (DOC 374 kb)
