# Supplementary material for: Methyl Jasmonate-Induced Monoterpenes in Scots Pine and Norway Spruce Tissues Affect Pine Weevil Orientation
Source: J Chem Ecol. 2016 Nov 28;42(12):1237–46. doi: 10.1007/s10886-016-0790-z (PMC5148791; doi:10.1007/s10886-016-0790-z)
Supplement: Supplementary file 1 — (DOC 374 kb) [file 10886_2016_790_MOESM1_ESM.doc]

**Electronic Supplementary Material of**

METHYL JASMONATE-INDUCED VOLATILES IN SCOTS PINE AND NORWAY SPRUCE TISSUES AFFECT PINE WEEVIL ORIENTATION

LINA LUNDBORG^1^, GÖRAN NORDLANDER^2^, NIKLAS BJÖRKLUND^2^, HENRIK NORDENHEM^2^, AND ANNA-KARIN BORG-KARLSON^1,3^

*^1^L. Lundborg (*), A.K. Borg-Karlson*

*KTH Royal Institute of Technology, School of Chemical Science*

*and Engineering, Department of Chemistry, Organic Chemistry,*

*SE-100 44, Stockholm, Sweden*

*e-mail: linalun@kth.se*

*^2^ G. Nordlander, N. Björklund, H. Nordenhem*

*Swedish University of Agricultural Sciences, Department of*

*Ecology, P.O. Box 7044, SE-750 07, Uppsala, Sweden*

*^3^A. K. Borg-Karlson*

*Tartu University, Institute of Technology, Division of Organic*

*Chemistry, Tartu 50411, Estonia*

* corresponding author *e-mail:* [*linalun@kth.se*](mailto:linalun@kth.se)


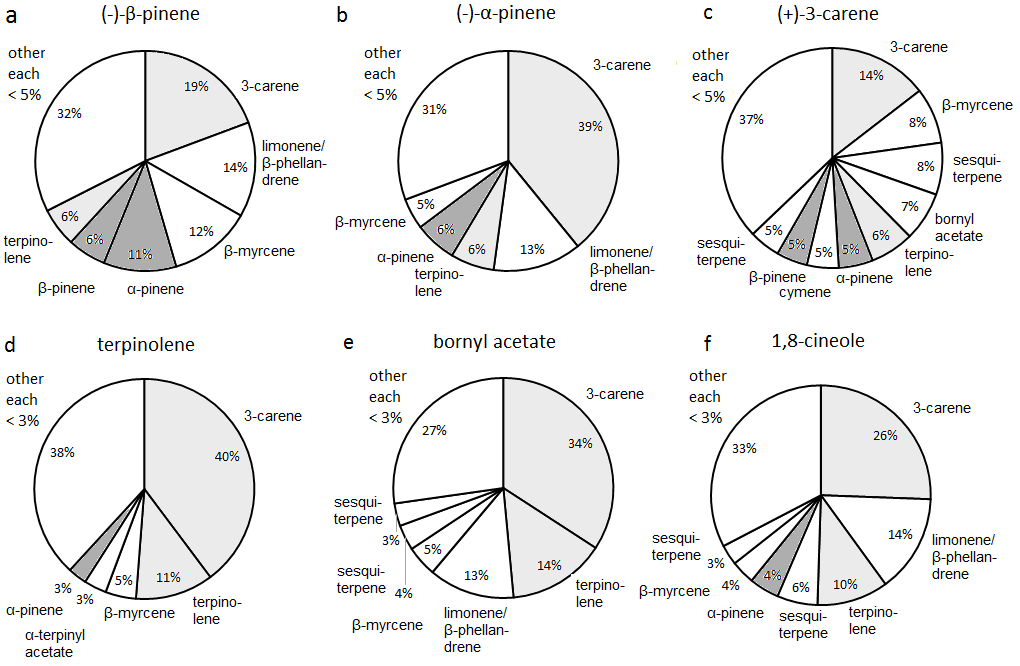


**Fig. 1** Relative proportions of volatiles emitted by the twigs from mature *Pinus sylvestris* trees used in the pine weevil bioassays. The dominant volatile in the twig emissions was 3-carene, with varying amounts of other monoterpenes. The shading shows biosynthetically related monoterpenes. In *Picea abies*, synthesis of 3-carene and terpinolene (light grey) and the (*-*) enantiomers of both β-pinene and α-pinene (dark grey) is catalyzed by different synthases^1^, similar to *Pinus* synthases^2^. Minor compounds (each accounting for <3 % or <5 % of the total amount) are grouped together in the other category. Emissions were collected and analyzed by solid-phase microextraction followed by gas-chromatography mass-spectrometry (SPME GC-MS). The SPME involved use of a PDMS/DVB fiber (65 µm film thickness, Stableflex, needle size 24 ga, Supelco) in a manual holder. A twig was cut to a length of 2.5 cm, pierced with a needle, and placed in a 35 ml beaker covered with aluminum foil. The pine emissions were collected by exposing the SPME fiber inside the beaker for 120 min. Instrument and instrument settings were the same as described for the chemical analysis of solvent extracts.

^1^ Zulak KG et al. Targeted proteomics using selected reaction monitoring reveals the induction of specific terpene synthases in a multi-level study of methyl jasmonate-treated Norway spruce (*Picea abies*) (2009) Plant J 6:1015-1030

^2^ Hall D et al. Transcriptome resources and functional characterization of monoterpene synthases for two host species of the mountain pine beetle, lodgepole pine (*Pinus contorta*) and jack pine (*Pinus banksiana*) BMC Plant Biol 1:1-14

| Table 1. Absolute amounts of quantified compounds in basal phloem and basal needle tissues of control and methyl jasmonate (MeJA)-treated *Pinus sylvestris* seedlings expressed in pentadecane equivalents (µg g^-1^ d.w.) ± SE. Abbreviations: uk=unknown, MT=monoterpene, ST=sesquiterpene, nd=not detected. | | | | | | | | | | | | |  |
| --- | --- | --- | --- | --- | --- | --- | --- | --- | --- | --- | --- | --- | --- |
| **Compound** | ***P. sylvestris* absolute amounts** | | | | | | | | | | | |  |
|  | | | Phloem | | | | Needles | | | | | |  |
|  | | Control | | | MeJA | | | | Control | | MeJA | |  |
| **Green leaf volatiles** | | | |  |  |  | | | |  | |  | |
| 1-methyl-cyclopentanol | | 3.2 ± 1.8 | | | 6.6 ± 1.3 | | | 2.5 ± 0.5 | | | 2.3 ± 0.3 | | |
| 3-hexanol | | 2.2 ± 0.2 | | | 2.1 ± 0.2 | | | 1.3 ± 0.2 | | | 1.7 ± 0.2 | | |
| 3-hexenal | | 0.2 ± 0.0 | | | 0.2 ± 0.0 | | | 4.1 ± 0.9 | | | 12.0 ± 5.5 | | |
| 3-methyl-cyclopentanol | | 3.0 ± 0.3 | | | 2.1 ± 1.3 | | | 3.5 ± 0.5 | | | 15.4 ± 3.1 | | |
| 2-hexenal | | nd | | | 1.5 ± 0.3 | | | 8.4 ± 2.1 | | | 31.9 ± 7.0 | | |
| 3-hexen-1-ol | | 0.3 ± 0.1 | | | 0.6 ± 0.1 | | | 3.1 ± 0.6 | | | 9.4 ± 1.1 | | |
|  | |  | | |  | | |  | | |  | | |
| **Monoterpenes** | |  | | |  | | |  | | |  | | |
| tricyclene | | 1.5 ± 0.5 | | | 0.9 ± 0.2 | | | 2.7 ± 0.5 | | | 3.9 ± 0.5 | | |
| α-thujene | | 9.1 ± 0.5 | | | 8.1 ± 0.6 | | | 11.5 ± 1.1 | | | 10.2 ± 0.8 | | |
| (-)-α-pinene | | 173.2 ± 47.4 | | | 138.6 ± 49.1 | | | 223.8 ± 59.9 | | | 191.3 ± 46.9 | | |
| (+)-α-pinene | | 481.2 ± 129.2 | | | 267.3 ± 39.3 | | | 711.7 ± 148.7 | | | 535.5 ± 83.3 | | |
| camphene | | 9.7 ± 2.3 | | | 5.9 ± 1.5 | | | 13.7 ± 2.7 | | | 10.8 ± 1.6 | | |
| sabinene | | 88.5 ± 10.9 | | | 66.8 ± 9.0 | | | 89.0 ± 10.9 | | | 83.4 ± 11.1 | | |
| (-)-β-pinene | | 33.7 ± 10.8 | | | 224.7 ± 99.1 | | | 24.4 ± 7.1 | | | 349.4 ± 100.1 | | |
| (+)-β-pinene | | 5.2 ± 0.9 | | | 5.0 ± 1.4 | | | 10.6 ± 4.8 | | | 8.9 ± 1.9 | | |
| β-myrcene | | 73.9 ± 10.0 | | | 60.3 ± 7.2 | | | 80.0 ± 8.2 | | | 81.4 ± 8.0 | | |
| α-phellandrene | | 4.6 ± 0.6 | | | 3.4 ± 0.4 | | | 4.2 ± 0.5 | | | 4.5 ± 0.6 | | |
| (+)-3-carene | | 2190.2 ± 271.7 | | | 1624.1 ± 206.2 | | | 2093.8 ± 231.7 | | | 2129.6 ± 303.8 | | |
| β-phellandrene | | 46.4 ± 8.2 | | | 40.0 ± 5.8 | | | 42.1 ± 5.7 | | | 52.0 ± 6.0 | | |
| (-)-limonene | | 29.0 ± 15.1 | | | 5.2 ± 0.8 | | | 20.4 ± 8.8 | | | 7.1 ± 0.9 | | |
| (+)-limonene | | 4.2 ± 1.3 | | | 2.6 ± 0.4 | | | 5.3 ± 1.4 | | | 4.1 ± 0.6 | | |
| 1,8-cineole | | 0.7 ± 0.3 | | | 10.0 ± 2.9 | | | 0.2 ± 0.1 | | | 3.1 ± 2.4 | | |
| *m/z* 93 | | 0.4 ± 0.1 | | | 2.7 ± 1.5 | | | 0.8 ± 0.2 | | | 0.6 ± 0.2 | | |
| ocimene | | 3.1 ± 1.1 | | | 2.5 ± 1.8 | | | 5.8 ± 1.9 | | | 1.9 ± 0.8 | | |
| *m/z* 77 93 | | 1.7 ± 0.2 | | | 1.2 ± 0.4 | | | 1.5 ± 0.2 | | | 1.6 ± 0.3 | | |
| uk MT 2 | | 31.1 ± 3.8 | | | 22.0 ± 4.4 | | | 26.9 ± 3.1 | | | 28.8 ± 4.1 | | |
| uk MT 3 | | 12.8 ± 1.7 | | | 10.1 ± 1.3 | | | 10.9 ± 1.3 | | | 11.1 ± 1.3 | | |
| terpinolene | | 281.4 ± 35.4 | | | 208.7 ± 30.1 | | | 220.0 ± 28.0 | | | 229.1 ± 31.3 | | |
| bornyl acetate | | 5.1 ± 2.2 | | | 3.3 ± 1.5 | | | 2.4 ± 1.3 | | | 1.5 ± 0.8 | | |
| uk MT 4 | | 22.2 ± 7.0 | | | 11.0 ± 2.7 | | | 10.4 ± 3.3 | | | 8.3 ± 1.9 | | |
| α-terpinyl acetate | | 68.4 ± 22.5 | | | 28.4 ± 9.2 | | | 32.3 ± 11.1 | | | 23.9 ± 6.0 | | |
|  | |  | | |  | | |  | | |  | | |
| **Aromatics** | |  | | |  | | |  | | |  | | |
| cymen-8-ol isomer 1 | | 2.4 ± 1.2 | | | 0.8 ± 0.3 | | | 4.3 ± 1.2 | | | 3.9 ± 2.4 | | |
| cymen-8-ol isomer 2 | | 2.0 ± 0.5 | | | 1.8 ± 0.5 | | | 2.6 ± 0.9 | | | 3.5 ± 2.0 | | |
| 2-phenylethanol | | 0.5 ± 0.1 | | | 5.8 ± 1.8 | | | 0.5 ± 0.1 | | | 17.6 ± 1.7 | | |
| methyl thymyl ether | | 6.3 ± 1.9 | | | 2.5 ± 0.3 | | | 2.8 ± 0.9 | | | 1.9 ± 0.4 | | |
| *m/z* 81 96 110 138 | | 4.2 ± 1.2 | | | 4.0 ± 1.1 | | | 6.4 ± 2.2 | | | 4.3 ± 0.8 | | |
|  | |  | | |  | | |  | | |  | | |
| **Sesquiterpenes** | |  | | |  | | |  | | |  | | |
| ylangene | | 4.8 ± 1.2 | | | 5.3 ± 2.3 | | | 2.6 ± 0.5 | | | 2.4 ± 0.7 | | |
| isoledene | | 3.3 ± 0.9 | | | 0.9 ± 0.2 | | | 3.3 ± 1.1 | | | 1.6 ± 0.3 | | |
| uk ST 1 | | 26.0 ± 10.8 | | | 5.9 ± 3.7 | | | 10.1 ± 4.4 | | | 3.1 ± 2.2 | | |
| β-caryophyllene | | 96.0 ± 20.2 | | | 84.8 ± 19.5 | | | 108.0 ± 13.3 | | | 121.3 ± 23.7 | | |
| *m/z* 105 | | 33.0 ± 13.6 | | | 1.8 ± 1.6 | | | 9.1 ± 2.5 | | | 0.9 ± 0.5 | | |
| β-farnesene | | 3.0 ± 1.4 | | | 1.2 ± 0.4 | | | 1.9 ± 0.5 | | | 1.3 ± 0.2 | | |
| *m/z* 161 189 204 | | 2.0 ± 0.7 | | | 0.6 ± 0.3 | | | 1.0 ± 0.2 | | | 0.3 ± 0.1 | | |
| α-humulene | | 15.5 ± 3.1 | | | 14.0 ± 3.2 | | | 17.5 ± 2.1 | | | 19.8 ± 3.8 | | |
| *m/z* 161 204 | | 2.4 ± 1.0 | | | 1.0 ± 0.5 | | | 3.6 ± 1.3 | | | 1.3 ± 0.5 | | |
| *m/z* 161 189 204 | | 2.9 ± 0.9 | | | 1.2 ± 0.5 | | | 2.1 ± 0.7 | | | 2.0 ± 0.8 | | |
| γ-elemene | | 91.0 ± 26.6 | | | 36.8 ± 9.2 | | | 86.4 ± 27.1 | | | 42.6 ± 9.0 | | |
| *m/z* 147 189 204 | | 3.3 ± 0.7 | | | 2.1 ± 1.2 | | | 2.9 ± 0.9 | | | 1.6 ± 0.3 | | |
| *m/z* 161 204 | | 14.7 ± 5.6 | | | 4.9 ± 1.8 | | | 15.2 ± 5.3 | | | 6.4 ± 1.9 | | |
| *m/z* 161 207 | | 15.1 ± 8.1 | | | 23.8 ± 12.8 | | | 5.4 ± 1.5 | | | 8.6 ± 3.9 | | |
| *m/z* 161 204 | | 24.6 ± 8.5 | | | 8.9 ± 2.1 | | | 21.7 ± 8.9 | | | 11.6 ± 3.3 | | |
| *m/z* 105 119 161 207 | | 2.3 ± 0.7 | | | 1.4 ± 0.9 | | | 2.2 ± 0.7 | | | 1.4 ± 0.4 | | |
| *m/z* 105 161 177 204 | | 2.7 ± 1.0 | | | 0.8 ± 0.3 | | | 2.8 ± 0.9 | | | 1.9 ± 0.5 | | |
| germacrene D-4-ol | | 324.8 ± 119.5 | | | 106.6 ± 22.6 | | | 322.4 ± 137.9 | | | 110.5 ± 22.0 | | |
| caryophyllene oxide | | 9.5 ± 3.8 | | | 1.6 ± 0.9 | | | 10.8 ± 2.6 | | | 8.4 ± 3.3 | | |
| *m/z* 119 137 | | 44.3 ± 6.5 | | | 68.6 ± 17.0 | | | 28.4 ± 6.6 | | | 25.8 ± 6.4 | | |
| *m/z* 161 194 204 | | 3.4 ± 1.0 | | | 1.6 ± 0.8 | | | 4.0 ± 1.1 | | | 3.2 ± 1.0 | | |
| *m/z* 95 148 121 161 204 | | 13.0 ± 7.2 | | | 2.7 ± 0.5 | | | 20.4 ± 7.5 | | | 14.9 ± 5.0 | | |

| Table 2. Absolute amounts of compounds in apical tissues of control and methyl jasmonate (MeJA)-treated *Pinus sylvestris* seedlings expressed in pentadecane equivalents (µg g^-1^ d.w.) ± SE. Abbreviations: uk=unknown, MT=monoterpene, ST=sesquiterpene. | | | | | | | | | |  |
| --- | --- | --- | --- | --- | --- | --- | --- | --- | --- | --- |
| **Compound** | | | ***P. sylvestris* absolute amounts** | | | | | | |  |
|  | Apical needles | | | | | | Shoot elongation zone | | |  |
|  | | Control | | MeJA |  | Control | | MeJA | | |
| **Green leaf volatiles** | |  | |  |  |  | |  | | |
| 1-methyl-cyclopentanol | | 3.0 ± 0.5 | | 4.1 ± 1.1 |  | 11.0 ± 2.7 | | | 10.5 ± 2.1 | |
| 3-hexanol | | 1.9 ± 0.3 | | 2.0 ± 0.5 |  | 3.2 ± 0.6 | | | 2.3 ± 0.3 | |
| 3-hexenal | | 21.4 ± 6.0 | | 13.7 ± 5.7 |  | 3.7 ± 1.3 | | | 6.7 ± 2.1 | |
| 3-methyl-cyclopentanol | | 12.6 ± 2.6 | | 16.2 ± 5.6 |  | 3.7 ± 1.0 | | | 5.2 ± 2.0 | |
| 2-hexenal | | 42.7 ± 8.5 | | 34.9 ± 10.9 |  | 1.8 ± 0.4 | | | 3.4 ± 0.7 | |
| 3-hexen-1-ol | | 0.2 ± 0.1 | | 3.6 ± 1.0 |  | 0.4 ± 0.1 | | | 1.3 ± 0.3 | |
|  | |  | |  |  |  | | |  | |
| **Monoterpenes** | |  | |  |  |  | | |  | |
| tricyclene | | 9.7 ± 1.8 | | 4.1 ± 1.2 |  | 3.7 ± 1.2 | | | 7.0 ± 4.0 | |
| α-thujene | | 3.3 ± 0.3 | | 6.0 ± 1.3 |  | 10.8 ± 2.9 | | | 20.6 ± 9.8 | |
| (-)-α-pinene | | 256.4 ± 74.0 | | 176.0 ± 36.5 |  | 476.0 ± 176.7 | | | 358.6 ± 73.1 | |
| (+)-α-pinene | | 760.7 ± 185.1 | | 708.6 ± 178.7 |  | 844.0 ± 282.5 | | | 1381.0 ± 657.6 | |
| camphene | | 40.3 ± 7.8 | | 20.2 ± 6.0 |  | 23.1 ± 7.0 | | | 40.8 ± 23.3 | |
| sabinene | | 45.5 ± 3.2 | | 90.2 ± 20.1 |  | 146.3 ± 38.2 | | | 144.5 ± 27.4 | |
| (-)-β-pinene | | 29.4 ± 5.4 | | 240.6 ± 74.5 |  | 93.5 ± 26.3 | | | 1074.6 ± 612.8 | |
| (+)-β-pinene | | 8.1 ± 1.7 | | 12.4 ± 4.0 |  | 11.6 ± 3.4 | | | 26.3 ± 11.6 | |
| β-myrcene | | 48.3 ± 4.4 | | 73.2 ± 19.5 |  | 132.0 ± 40.5 | | | 165.4 ± 30.3 | |
| α-phellandrene | | 2.0 ± 0.3 | | 4.5 ± 0.9 |  | 7.7 ± 2.0 | | | 11.1 ± 2.9 | |
| (+)-3-carene | | 983.5 ± 73.0 | | 1790.6 ± 386.4 |  | 3339.5 ± 846.1 | | | 4188.3 ± 919.4 | |
| β-phellandrene | | 22.8 ± 2.6 | | 61.1 ± 16.6 |  | 107.3 ± 29.6 | | | 155.7 ± 50.1 | |
| (-)-limonene | | 5.1 ± 1.2 | | 6.9 ± 1.4 |  | 202.8 ± 84.0 | | | 22.6 ± 10.7 | |
| (+)-limonene | | 5.6 ± 1.2 | | 4.8 ± 0.9 |  | 15.0 ± 4.8 | | | 9.7 ± 2.7 | |
| 1,8-cineole | | 0.5 ± 0.2 | | 0.4 ± 0.2 |  | 0.8 ± 0.3 | | | 1.8 ± 1.0 | |
| uk MT 2 | | 9.1 ± 2.2 | | 16.0 ± 4.1 |  | 49.6 ± 14.5 | | | 59.9 ± 12.4 | |
| terpinolene | | 116.3 ± 8.9 | | 236.2 ± 52.1 |  | 481.5 ± 130.6 | | | 548.3 ± 93.3 | |
| bornyl acetate | | 3.9 ± 1.1 | | 2.7 ± 0.7 |  | 8.4 ± 4.3 | | | 15.8 ± 8.2 | |
| uk MT 4 | | 2.9 ± 1.0 | | 11.4 ± 3.4 |  | 13.8 ± 4.3 | | | 26.1 ± 6.6 | |
| α-terpinyl acetate | | 9.7 ± 3.9 | | 40.8 ± 12.4 |  | 37.8 ± 15.4 | | | 86.2 ± 23.0 | |
|  | |  | |  |  |  | | |  | |
| **Aromatics** | |  | |  |  |  | | |  | |
| cymen-8-ol isomer 1 | | 0.2 ± 0.2 | | 5.4 ± 3.6 |  | 1.2 ± 0.7 | | | 21.5 ± 17.5 | |
| cymen-8-ol isomer 2 | | 0.4 ± 0.1 | | 3.0 ± 1.9 |  | 3.9 ± 2.7 | | | 12.5 ± 9.9 | |
| 2-phenylethanol  methyl thymyl ether | | 0.3 ± 0.0  0.5 ± 0.2 | | 4.5 ± 1.1  1.7 ± 0.2 |  | 0.7 ± 0.2  6.1 ± 3.1 | | | 7.7 ± 3.0  8.4 ± 5.0 | |
| *m/z* 81 96 110 138 | | 0.3 ± 0.1 | | 2.6 ± 0.8 |  | 13.4 ± 6.6 | | | 15.5 ± 4.2 | |
|  | |  | |  |  |  | | |  | |
| **Sesquiterpenes** | |  | |  |  |  | | |  | |
| ylangene | | 1.3 ± 0.2 | | 3.1 ± 1.0 |  | 6.9 ± 2.7 | | | 9.9 ± 2.6 | |
| isoledene | | 2.2 ± 0.4 | | 2.4 ± 0.7 |  | 3.2 ± 1.9 | | | 3.4 ± 0.5 | |
| uk ST 1 | | 0.1 ± 0.0 | | 1.1 ± 0.8 |  | 29.9 ± 17.5 | | | 16.3 ± 9.3 | |
| β-caryophyllene | | 153.6 ± 23.8 | | 205.6 ± 43.4 |  | 260.7 ± 91.0 | | | 339.2 ± 82.5 | |
| *m/z* 105 | | 1.5 ± 0.9 | | 1.4 ± 1.1 |  | 68.0 ± 34.0 | | | 5.1 ± 4.3 | |
| β-farnesene | | 1.6 ± 0.3 | | 2.0 ± 0.6 |  | 3.8 ± 1.5 | | | 4.2 ± 0.7 | |
| *m/z* 161 189 204 | | 0.1 ± 0.0 | | 0.5 ± 0.3 |  | 3.9 ± 2.5 | | | 2.6 ± 0.4 | |
| α-humulene | | 26.0 ± 3.9 | | 34.7 ± 7.3 |  | 41.9 ± 15.6 | | | 56.5 ± 13.6 | |
| *m/z* 161 204 | | 3.3 ± 1.0 | | 3.3 ± 1.6 |  | 4.4 ± 2.0 | | | 2.2 ± 0.5 | |
| *m/z* 161 189 204 | | 1.5 ± 0.4 | | 1.8 ± 0.7 |  | 4.9 ± 2.3 | | | 7.4 ± 4.5 | |
| γ-elemene | | 45.8 ± 10.5 | | 31.2 ± 4.8 |  | 213.2 ± 137.4 | | | 81.2 ± 19.7 | |
| *m/z* 147 189 204 | | 1.5 ± 0.4 | | 15.6 ± 14.8 |  | 32.1 ± 28.2 | | | 4.4 ± 2.5 | |
| *m/z* 161 204 | | 14.3 ± 3.3 | | 11.2 ± 5.4 |  | 36.4 ± 19.0 | | | 5.6 ± 1.8 | |
| *m/z* 161 207 | | 0.5 ± 0.2 | | 13.7 ± 6.5 |  | 16.8 ± 8.5 | | | 31.1 ± 13.3 | |
| *m/z* 161 204 | | 16.9 ± 4.0 | | 15.4 ± 5.0 |  | 48.4 ± 30.5 | | | 32.0 ± 14.1 | |
| *m/z* 105 119 161 207 | | 0.8 ± 0.3 | | 0.7 ± 0.3 |  | 3.9 ± 2.3 | | | 2.5 ± 0.6 | |
| *m/z* 105 161 177 204 | | 2.1 ± 0.7 | | 1.9 ± 0.7 |  | 4.4 ± 2.2 | | | 3.1 ± 0.9 | |
| germacrene D-4-ol | | 306.9 ± 89.7 | | 264.9 ± 87.6 |  | 516.9 ± 305.3 | | | 197.9 ± 37.1 | |
| caryophyllene oxide | | 1.2 ± 0.3 | | 8.7 ± 3.6 |  | 5.7 ± 1.9 | | | 37.9 ± 23.3 | |
| *m/z* 119 137 161 194 204 | | 38.0 ± 5.9 | | 41.5 ± 9.8 |  | 58.0 ± 15.4 | | | 96.0 ± 27.3 | |
| *m/z* 119 137 161 194 204 | | 2.9 ± 0.7 | | 2.8 ± 0.4 |  | 3.3 ± 1.3 | | | 7.3 ± 2.2 | |
| *m/z* 95 148 121 161 204 | | 19.4 ± 4.2 | | 14.1 ± 3.0 |  | 15.2 ± 8.2 | | | 14.5 ± 6.8 | |

| Table 3. Absolute amounts of quantified compounds in phloem of control and methyl jasmonate (MeJA)-treated *Picea abies* seedlings expressed in pentadecane equivalents (µg g^-1^ d.w.) ± SE. Abbreviations: uk=unknown, MT=monoterpene, nd=not detected. | | | | | |
| --- | --- | --- | --- | --- | --- |
| **Compound** | ***P. abies* absolute amounts** | | | | |
|  | Lower phloem | | Upper phloem | | |
|  | Control | MeJA | | Control | MeJA |
| **Green leaf volatiles** |  |  | |  |  |
| 1-methyl-cyclopentanol | 46.5 ± 1.8 | 42.9 ± 2.0 | | 58.5 ± 2.9 | 48.3 ± 2.0 |
| 3-hexanol | 7.0 ± 0.2 | 6.7 ± 0.3 | | 8.8 ± 0.4 | 7.3 ± 0.3 |
| 3-hexenal | 8.8 ± 0.2 | 9.2 ± 0.4 | | 11.3 ± 0.7 | 11.6 ± 0.9 |
| 3-methyl-cyclopentanol | 10.0 ± 0.3 | 9.8 ± 0.6 | | 10.8 ± 1.5 | 10.9 ± 0.5 |
| 2-hexenal | nd | 1.0 ± 0.3 | | 0.5 ± 0.3 | 2.9 ± 0.6 |
| 3-hexen-1-ol | nd | 3.6 ± 0.9 | | 1.0 ± 0.7 | 3.7 ± 0.7 |
|  |  |  | |  |  |
| **Monoterpenes** |  |  | |  |  |
| tricyclene | 30.7 ± 8.5 | 27.4 ±6.7 | | 12.9 ± 1.3 | 28.4 ± 4.8 |
| α-thujene | 9.3 ± 2.2 | 5.8 ± 1.5 | | 6.8 ± 3.0 | 11.2 ± 3.7 |
| (*-*)-α-pinene | 2122.0 ± 393.4 | 1962.4 ± 392.0 | | 1239.1 ±148.2 | 2410.1 ± 554.6 |
| (*+*)-α-pinene | 253.3 ± 40.2 | 388.0 ± 142.9 | | 109.0 ± 16.5 | 401.3 ± 107.6 |
| camphene | 231.7 ± 67.5 | 197.7 ± 54.2 | | 97.4 ± 10.0 | 192.5 ± 31.7 |
| sabinene | 101.1 ± 33.8 | 74.3 ± 24.0 | | 117.6 ± 44.5 | 136.3 ± 37.9 |
| (*-*)-β-pinene | 2200.3 ± 592.1 | 2904.9 ± 720.8 | | 1267.5 ± 215.9 | 3731.0 ± 738.9 |
| (*+*)-β-pinene | 41.6 ± 6.9 | 45.5 ± 9.0 | | 24.3 ± 2.2 | 57.5 ± 9.9 |
| β-myrcene | 314.4 ± 55.9 | 372.9 ± 76.8 | | 278.0 ± 41.8 | 474.8 ± 120.3 |
| α-phellandrene | 30.7 ± 8.5 | 27.9 ± 5.8 | | 15.1 ± 2.6 | 30.2 ± 4.8 |
| (+)-3-carene | 1007.6 ± 341.5 | 624.6 ± 255.1 | | 1075.3 ± 409.2 | 1261.1 ± 345.0 |
| uk MT 1 | 7.7 ± 2.4 | 4.9 ± 1.5 | | 6.1 ± 3.0 | 10.3 ± 2.6 |
| (-)-limonene | 644.3 ± 212.6 | 378.9 ± 114.8 | | 846.1 ± 352.9 | 741.9 ± 243.7 |
| (+)-limonene | 470.5 ± 443.2 | 36.9 ± 7.7 | | 27.9 ± 5.9 | 232.3 ± 163.5 |
| β-phellandrene | 1610.0 ± 235.3 | 2116.5 ± 418.0 | | 1158.4 ± 110.1 | 2674.8 ± 625.0 |
| ocimene | 0.5 ± 0.2 | 1.4 ± 0.3 | | 0.5 ± 0.3 | 1.4 ± 0.6 |
| uk MT 2 | 18.5 ± 5.8 | 12.7 ± 4.1 | | 18.1 ± 7.1 | 19.4 ± 6.1 |
| uk MT 3 | 5.4 ± 1.9 | 3.0 ± 1.2 | | 4.8 ± 1.9 | 6.0 ± 1.2 |
| terpinolene | 197.9 ± 53.8 | 140.4 ± 38.2 | | 170.3 ± 60.4 | 214.6 ± 46.1 |
| 1,8-cineole | 2.5 ± 1.6 | 15.2 ± 5.6 | | 1.9 ± 0.5 | 12.4 ± 3.7 |
| cis-β-terpineol | 5.8 ± 3.8 | 6.3 ± 3.8 | | 0.6 ± 0.1 | 2.0 ± 0.4 |
| endo-borneol | 10.7 ± 3.1 | 7.6 ± 1.3 | | 4.2 ± 0.4 | 6.3 ± 1.1 |
| 1-terpinen-4-ol | 1.0 ± 0.5 | 3.8 ± 1.2 | | 0.3 ± 0.0 | 1.5 ± 0.5 |
| fenchyl acetate | 0.7 ± 0.4 | 1.5 ± 1.1 | | 0.2 ± 0.0 | 0.5 ± 0.2 |
| bornyl acetate | 134.8 ± 51.0 | 110.2 ± 25.9 | | 74.7 ± 11.0 | 182.7 ± 25.6 |
| camphor | 10.3 ± 4.8 | 8.9 ± 7.2 | | 1.9 ± 0.5 | 3.2 ± 1.0 |
| linalool | 3.1 ± 0.8 | 28.4 ± 9.4 | | 0.6 ± 0.1 | 11.5 ± 3.2 |
|  |  |  | |  |  |
| **Aromatics** |  |  | |  |  |
| estragole | 7.1 ± 3.1 | 18.7 ± 11.2 | | 0.9 ± 0.2 | 6.3 ± 2.6 |
| methyl thymyl ether | 77.3 ± 19.1 | 75.9 ± 22.1 | | 35.2 ± 7.5 | 70.8 ± 16.9 |
|  |  |  | |  |  |
| **Sesquiterpenes** |  |  | |  |  |
| α-longipinene | 170.6 ± 64.9 | 157.3 ± 42.7 | | 113.9 ± 33.9 | 211.4 ± 80.7 |
| cyclosativene | 7.8 ± 2.5 | 12.3 ± 6.0 | | 5.1 ± 1.0 | 11.4 ± 5.3 |
| ylangene | 20.1 ± 6.1 | 22.8 ± 5.7 | | 11.9 ± 2.6 | 24.2 ± 5.1 |
| isoledene | 64.2 ± 17.5 | 83.3 ± 22.0 | | 36.7 ± 10.5 | 81.0 ± 23.0 |
| β-caryophyllene | 46.0 ± 13.6 | 62.1 ± 29.8 | | 40.8 ± 14.2 | 49.4 ± 11.6 |
| β-farnesene | 49.5 ± 14.2 | 42.0 ± 8.8 | | 34.0 ± 6.5 | 64.1 ± 19.3 |
| α-humulene | 48.0 ± 13.9 | 31.7 ± 12.6 | | 26.6 ± 10.0 | 24.3 ± 10.2 |
| cedrene | 13.7 ± 4.9 | 6.1 ± 3.2 | | 27.0 ± 12.1 | 7.9 ± 2.6 |
| δ-cadinene | 31.2 ± 10.4 | 47.0 ± 14.9 | | 13.8 ± 4.9 | 52.3 ± 15.7 |
| 1,3,8-p-menthatriene | 2.3 ± 0.6 | 4.0 ± 1.0 | | 2.2 ± 0.7 | 4.5 ± 1.0 |
| germacrene D-4-ol | 69.8 ± 15.2 | 45.2 ± 9.0 | | 25.3 ± 5.3 | 70.7 ± 17.2 |

| Table 4. Absolute amounts of quantified compounds in needle tissues of control and methyl jasmonate (MeJA)-treated *Picea abies* seedlings expressed in pentadecane equivalents (µg g^-1^ d.w.) ± SE. Abbreviations: uk=unknown, MT=monoterpene. | | | | |
| --- | --- | --- | --- | --- |
| **Compound** | ***P. abies* absolute amounts** | | | |
|  | Needles | | Shoot tip | |
|  | Control | MeJA | Control | MeJA |
| **Green leaf volatiles** |  |  |  |  |
| 1-methyl-cyclopentanol | 10.8 ± 1.8 | 9.8 ± 1.4 | 17.1 ± 1.2 | 19.4 ± 1.2 |
| 3-hexanol | 2.9 ± 0.4 | 3.3 ± 0.6 | 5.2 ± 0.4 | 6.5 ± 0.3 |
| 3-hexenal | 8.6 ± 1.1 | 20.3 ± 5.6 | 18.0 ± 3.6 | 22.0 ± 2.7 |
| 3-methyl-cyclopentanol | 5.7 ± 0.6 | 4.4 ± 0.3 | 5.7 ± 0.2 | 6.7 ± 0.4 |
| 2-hexenal | 13.0 ± 3.2 | 29.2 ± 6.8 | 40.7 ± 8.4 | 47.2 ± 6.0 |
| 3-hexen-1-ol | 7.5 ± 1.4 | 12.3 ± 1.6 | 2.6 ± 1.0 | 0.6 ± 0.5 |
|  |  |  |  |  |
| **Monoterpenes** |  |  |  |  |
| tricyclene | 232.9 ± 19.9 | 222.1 ± 15.0 | 241.9 ± 11.1 | 294.0 ± 13.9 |
| α-thujene | 4.6 ± 0.9 | 6.1 ± 0.9 | 10.6 ± 2.0 | 18.7 ± 2.0 |
| (-)-α-pinene | 887.7 ± 96.0 | 1047.6 ± 107.6 | 987.9 ± 39.3 | 1708.6 ± 95.1 |
| (+)-α-pinene | 325.6 ± 66.7 | 206.0 ± 42.0 | 237.1 ± 39.6 | 234.1 ± 35.8 |
| camphene | 2077.5 ± 238.0 | 1824.8 ± 121.3 | 2075.6 ± 97.1 | 2226.8 ± 32.6 |
| sabinene | 109.2 ± 19.7 | 135.2 ± 38.9 | 138.9 ± 17.8 | 143.6 ± 41.0 |
| (*-*)-β-pinene | 143.7 ± 36.7 | 300.7 ± 67.0 | 349.5 ± 160.0 | 999.4 ± 87.1 |
| (*+*)-β-pinene | 41.2 ± 9.9 | 24.2 ± 5.8 | 40.1 ± 11.4 | 33.1 ± 5.7 |
| β-myrcene | 356.1 ± 64.4 | 285.2 ± 45.1 | 425.9 ± 58.1 | 486.3 ± 74.2 |
| α-phellandrene | 11.7 ± 1.8 | 12.9 ± 1.3 | 22.7 ± 2.8 | 31.8 ± 2.5 |
| (*+*)-3-carene | 14.5 ± 4.7 | 53.0 ± 25.6 | 33.2 ± 7.7 | 33.8 ± 11.8 |
| uk MT 1 | 2.0 ± 0.3 | 2.1 ± 0.3 | 4.9 ± 0.7 | 5.7 ± 0.9 |
| (-)-limonene | 966.7 ± 221.3 | 678.9 ± 155.8 | 1209.6 ± 210.8 | 1178.4 ± 243.7 |
| (+)-limonene | 212.2 ± 27.1 | 161.6 ± 13.8 | 197.4 ± 19.2 | 187.5 ± 13.4 |
| β-phellandrene | 162.0 ± 34.9 | 326.5 ± 68.2 | 222.0 ± 52.4 | 595.8 ± 59.2 |
| ocimene | 35.6 ± 16.7 | 35.0 ± 13.5 | 63.1 ± 32.6 | 119.6 ± 53.0 |
| uk MT 2 | 3.5 ± 0.4 | 7.2 ± 2.9 | 2.0 ± 1.4 | 8.4 ± 2.1 |
| uk MT 3 | 2.2 ± 0.8 | 5.7 ± 2.4 | 2.2 ± 0.6 | 4.9 ± 2.0 |
| terpinolene | 69.8 ± 13.8 | 40.6 ± 5.9 | 104.3 ± 15.4 | 107.2 ± 11.4 |
| 1,8-cineole | 703.0 ± 134.4 | 799.9 ± 197.0 | 852.2 ± 97.3 | 792.2 ± 198.8 |
| cis-β-terpineol | 44.3 ± 6.5 | 37.4 ± 10.9 | 40.5 ± 3.6 | 33.1 ± 9.4 |
| endo-borneol | 139.2 ± 36.9 | 91.0 ± 8.7 | 140.2 ± 46.3 | 216.9 ± 54.3 |
| 1-terpinen-4-ol | 21.5 ± 3.7 | 24.1 ± 4.8 | 32.8 ± 3.7 | 23.7 ± 5.1 |
| fenchyl acetate | 16.8 ± 4.9 | 5.9 ± 2.3 | 15.0 ± 6.0 | 18.0 ± 5.2 |
| bornyl acetate | 6518.0 ± 811.9 | 5007.2 ± 314.1 | 6183.6 ± 700.9 | 5150.2 ± 351.4 |
| camphor | 103.7 ± 48.9 | 14.7 ± 11.3 | 247.3 ± 90.8 | 322.0 ± 214.8 |
| linalool | 56.0 ± 8.8 | 46.5 ± 11.0 | 58.1 ± 6.0 | 48.3 ± 11.8 |
|  |  |  |  |  |
| **Aromatics** |  |  |  |  |
| estragole | 20.1 ± 7.6 | 26.6 ± 8.7 | 17.7 ± 6.7 | 25.5 ± 8.0 |
| methyl thymyl ether | 4.4 ± 1.3 | 7.3 ± 2.3 | 3.7 ± 0.6 | 5.0 ± 1.4 |
|  |  |  |  |  |
| **Sesquiterpenes** |  |  |  |  |
| α-longipinene | 31.4 ± 6.7 | 50.6 ± 11.4 | 34.4 ± 6.7 | 71.9 ± 14.0 |
| cyclosativene | 21.1 ± 20.1 | 1.8 ± 0.8 | 1.9 ± 0.6 | 2.7 ± 0.7 |
| ylangene | 7.5 ± 5.8 | 1.5 ± 0.1 | 2.1 ± 0.2 | 2.9 ± 0.8 |
| isoledene | 3.5 ± 1.0 | 7.1 ± 2.1 | 7.6 ± 1.6 | 11.8 ± 2.4 |
| β-caryophyllene | 100.7 ± 21.7 | 59.6 ± 12.5 | 162.5 ± 34.0 | 108.2 ± 24.6 |
| β-farnesene | 23.4 ± 4.8 | 26.7 ± 4.1 | 24.2 ± 4.1 | 27.2 ± 4.6 |
| α-humulene | 99.7 ± 21.4 | 57.1 ± 12.5 | 161.9 ± 33.0 | 100.7 ± 25.3 |
| cedrene | 0.8 ± 0.2 | 0.9 ± 0.5 | 2.2 ± 0.8 | 2.5 ± 1.1 |
| δ-cadinene | 9.3 ± 1.3 | 10.1 ± 1.8 | 20.8 ± 1.5 | 26.2 ± 3.5 |
| 1,3,8-p-menthatriene | 11.3 ± 1.6 | 15.0 ± 1.8 | 3.7 ± 0.4 | 3.9 ± 0.3 |
